# In Vivo Prostate Cancer Modelling: From the Pre-Clinical to the Clinical Setting

**DOI:** 10.3390/life16010111

**Published:** 2026-01-13

**Authors:** Elisabete Nascimento-Gonçalves, Tiago Azevedo, Catarina Medeiros, Ana I. Faustino-Rocha

**Affiliations:** 1 Centre for the Research and Technology of Agro-Environmental and Biological Sciences (CITAB), Institute for Innovation, Capacity Building and Sustainability of Agri-Food Production (Inov4Agro), University of Trás-os-Montes and Alto Douro (UTAD), 5000-801 Vila Real, Portugal; elisabete.nascimento.g@gmail.com (E.N.-G.); tiagoaazevedo99@gmail.com (T.A.); catarinamedeiros2508@outlook.com (C.M.); 2 Department of Zootechnics, School of Sciences and Technology, University of Évora, 7004-516 Évora, Portugal; 3 Comprehensive Health Research Center (CHRC), University of Évora, 7004-516 Évora, Portugal

**Keywords:** carcinogenesis, drug development, rat, zebrafish, preclinical research, prostate

## Abstract

Prostate cancer (PCa) remains one of the most prevalent malignancies in men and a leading cause of cancer-related mortality worldwide. Over the last century, PCa modelling has evolved from basic cell-based to more complex systems. Despite this, the clinical translation of research findings is limited by the constraints of current preclinical models. In this review, rat and zebrafish models are highlighted due to their long-standing and emerging translational relevance, respectively. Rat models have played a pivotal role in understanding carcinogenesis and supporting the preclinical evaluation of drugs currently approved for clinical use, such as antiandrogens and androgen-deprivation agents. In parallel, zebrafish models are increasingly recognized as powerful complementary tools for studying tumor biology, metastasis, and drug response, offering unique advantages for high-throughput and personalized medicine approaches. We summarize historical milestones, current advances, and translational perspectives, emphasizing how combining multiple model systems can bridge the gap between molecular research and clinical application. Collectively, the development and refinement of these models represent essential steps toward more predictive and ethically responsible PCa research.

## 1. Introduction

Prostate cancer (PCa) represents a major global health burden, ranking as the second most frequently diagnosed cancer and the fifth leading cause of cancer-related mortality among men worldwide [1]. In 2022, approximately 1.46 million new cases and 396,000 deaths were reported, accounting for 7.3% of all new cancer diagnoses and 4.1% of global cancer mortality [2,3]. The disease predominantly affects men between 45 and 60 years of age and displays marked geographic variation, with incidence rates nearly six times higher in Western countries compared to non-Western regions [1,4]. This disparity reflects the combined influence of genetic predisposition, racial background, lifestyle factors, and differences in healthcare access and diagnostic practices [5].

Clinically, PCa is highly heterogeneous, spanning from indolent tumors and slow growing tumors to highly aggressive variants with early metastatic potential [6]. Its progression is influenced by genomic alterations and tumor–microenvironment interactions. Host-related factors such as age, hereditary and germline genetic alterations, ethnicity, immune status, and metabolic state also play critical roles [1,7,8,9]. This complexity limits early diagnosis, prognostic stratification, and the development of universally effective therapies [10,11,12]. Current therapeutic strategies include radical prostatectomy, radiotherapy, androgen-deprivation therapy and chemotherapy. Emerging approaches include prostate-specific membrane antigen (PSMA)-targeted radioligand therapy, immunotherapy, and poly ADP-ribose polymerase inhibitors [13,14,15,16]. These strategies are tailored according to disease stage, histological classification, and tumor aggressiveness [17,18]. Nevertheless, therapeutic decision-making in advanced PCa remains challenging due to its multifocality, anatomical variability, and heterogeneous growth patterns [10,11,12]. Current therapies also show limited long-term effectiveness, as many patients eventually develop resistance or recurrence [19,20].

Despite significant advances in molecular biology and imaging technologies, the translation of PCa research into consistent clinical benefit remains limited [21]. This gap underscores the importance of robust preclinical models capable of recapitulating the biological and clinical complexity of human PCa. A wide spectrum of models have been developed, from cell lines to more complex in vivo systems, each contributing to the study of molecular mechanisms and drug development [22]. In vitro models, particularly immortalized cell lines such as LNCaP, PC-3, and DU145, have been pivotal for elucidating molecular pathways and oncogenic mechanisms. They also allow high-throughput drug screening with minimal ethical concerns [23,24]. However, these systems cannot reproduce the complex tumor microenvironment, where interactions among cancer cells, stromal and immune components, and systemic factors influence disease initiation and progression [23,25]. Consequently, in vivo models provide a more physiologically relevant context and remain indispensable for investigating disease anatomy, physiology, pathogenesis, molecular mechanisms and therapeutic response [26]. Rodents, especially rats (*Rattus norvegicus*) and mice (*Mus musculus*), are the most widely used in PCa research due to advantages such as ease of handling and physiological and genetic similarities to humans [27]. Notably, the dorsolateral prostate of these species resembles the human prostate in development, function, and susceptibility to carcinogens, reinforcing their utility for modeling human disease [28].

Thus, this review provides an overview of the evolution and variety of PCa models, emphasizing their complementary contributions to translational research. Special attention is given to the role of rat models in drug development and the emerging potential of zebrafish models as versatile, high-throughput platforms that complement mammalian systems and support the advancement of precision oncology.

This review provides an overview of the evolution and variety of prostate cancer (PCa) models, highlighting their complementary roles in translational research. It gives special attention to rat models, documenting their significant contributions to drug development and Food and Drug Administration approval of prostate cancer therapies, offering a valuable and often overlooked perspective. The review also explores the emerging potential of zebrafish models as versatile, high-throughput platforms that complement mammalian systems, supporting the advancement of precision oncology while addressing their current biological and methodological limitations.

## 2. Prostate Cancer Modelling: From Traditional to Emerging Models

The biological and clinical complexity of PCa has driven the development of diverse experimental models capable of reproducing specific aspects of the disease. Over the past decades, researchers have been established a broad spectrum of in vitro and in vivo models, each offering unique strengths but also inherent limitations [29]. Preclinical approaches include computational, in vitro, and in vivo systems. In vitro approaches encompassed computational tools, 2D and 3D cell culture systems and microfluidic devices, whereas in vivo models range from rodents to chicken chorioallantoic membrane (CAM) and zebrafish embryo systems [30,31]. No single model faithfully replicates the full spectrum of human PCa. Therefore, combining multiple complementary systems has become an essential strategy for dissecting molecular mechanisms, studying tumor–host interactions, and enhancing the predictive value of preclinical findings [32,33]. Traditional approaches, including cell lines and chemically or hormonally induced rodent models, have provided foundational insights, while recent technological advances have led to the emergence of complex models such as organoids, patient-derived xenografts (PDXs), genetically engineered rodents, and organ-on-a-chip platforms [22,24,34]. In this review, rat and zebrafish models are highlighted due to their long-standing and emerging translational relevance, respectively, but they are contextualized within a broader landscape of traditional and next-generation platforms.

### 2.1. Tracing the Evolution of Prostate Cancer Models

**Figure 1:**
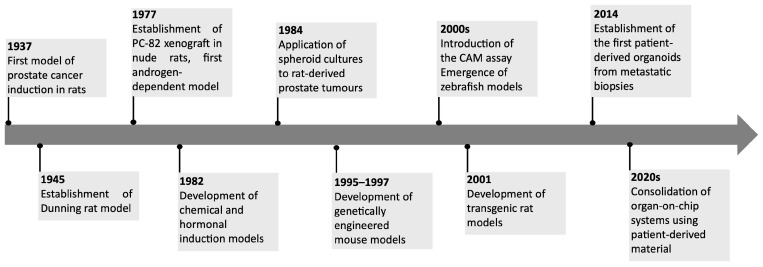
Timeline highlighting important milestones in the use of animal models for prostate cancer research.

The development of PCa models has progressed through several historical milestones (Figure 1), that reflect advances in cancer biology and experimental methodology. The first reported animal model of PCa dates to 1937 when Moore and Melchionna induced prostate carcinoma in rats through the injection of 1:2-benzpyrene into anterior prostate [35]. However, this model could not develop metastasis, which constituted a limitation. A major advancement occurred in 1945 when Dunning and colleagues produced methylcholanthrene-induced tumors in Fischer and AxC 9935 rats, generating lesions with metastatic potential and thus more closely resembling human disease [36]. Pollard and colleagues, in 1982, developed a combined chemical and hormonal induction protocol using *N*-methyl-*N*-nitrosourea and hormonal treatment in Lobund-Wistar rats [37]. This model, which recapitulates several hallmarks of human PCa, became one of the most widely used systems for studying carcinogenesis and tumor progression [38,39].

Parallel to these developments, the establishment of immortalized human PCa cell lines in the 1970s, including LNCaP, PC-3 and DU145, provided simple and reproducible tools for studying androgen signaling, metastatic potential, and drug response [40,41]. Nevertheless, conventional cell culture systems are inherently limited in reproducing the intricate cell–cell and cell–matrix interactions that define the tumor microenvironment, thereby restricting their translational relevance [42,43].

The late 1970s marked another turning point with the introduction of xenograft models, enabling the growth of human PCa tissues in immunodeficient mice [44]. The PC-82 xenograft, in 1987, was the first androgen-dependent model, followed by additional androgen-independent variants [44]. In 1996 were established seven further xenograft models directly from human prostate tumors, implanted in athymic nude mice [45]. These faithfully preserved the histopathological features of the donor tumors, including androgen dependence or independence.

Spheroids and organoids better mimic the architecture and microenvironment of prostate tumors, thereby providing greater translational relevance [46,47]. These models bridged the gap between reductionist 2D systems and animal models, offering new opportunities for translational drug discovery. Spheroid models were first applied to PCa research in the 1984 using rat-derived tumors [48], and were subsequently adapted for human PCa research when Ballangrud and colleagues [49] successfully generated spheroids from the LNCaP prostate cancer cell line. Organoids derived from patient biopsies or surgical specimens represent a promising tool for personalized medicine, as they retain molecular signatures and treatment responses observed in the donor [50,51]. Moreover, co-culture systems incorporating fibroblasts, endothelial cells, or immune cells have been developed to better mimic the tumor microenvironment [52]. The establishment of patient-derived prostate cancer organoids marked a significant milestone in 2014, when Gao et al. successfully cultured three-dimensional structures from metastatic biopsies and circulating tumor cells [53]. These organoids retained key molecular signatures of the original tumors and provided a new, physiologically relevant platform for studying prostate cancer heterogeneity and therapeutic responses in vitro.

The development of genetically engineered rodent models in the 1990s allowed the study of many aspects of cancer biology, including but not limited to mechanisms of sensitivity and resistance to drug treatment, oncogene cooperation, early detection, and metastasis. The transgenic adenocarcinoma of the mouse prostate (TRAMP) model was developed in 1995–1997. It used the minimal rat probasin (PB) promoter to target the large T SV40 virus expression and small t oncoproteins in the secretory epithelial cells [54]. This model has been used widely in PCa to study angiogenesis and to validate genes involved in PCa, for the testing and discovery of new drugs [31]. The PTEN model is also among the most frequently used, since PTEN is one of the most tumor-suppressor genes altered in the early events of PCa development [55]. The first PTEN knockout mouse was created in 1998 by generating a null mutation in the PTEN gene and, afterward, studies showed that PTEN is a tumor suppressor that is essential for embryonic development [56]. Similarly to the mice, in 2001, Makoto and colleagues created a transgenic rat prostate cancer model, named “transgenic rat with adenocarcinomas of the prostate (TRAP)”, using the probasin gene promoter and the Simian Virus-40 T (SV40-T) antigen in the genetic background of Sprague-Dawley rats [57].

In parallel, alternative in vivo models also emerged during this period, including the CAM assay and zebrafish embryo models. The CAM assay gained traction from the year 2000 as a platform to study tumor growth, angiogenesis, and metastasis in a highly vascularized environment [58,59]. Around the same period, zebrafish embryo models began to be applied to PCa, exploiting their optical transparency and genetic tractability to investigate cell invasion, angiogenesis, and drug screening [60].

Emerging platforms such as microfluidic devices and organ-on-a-chip systems represent a further refinement of 3D culture, allowing dynamic control of fluid flow, nutrient gradients and cell–cell interactions, thereby more closely reproducing the prostate tumor microenvironment [34,61,62]. Despite their promise, organ-on-a-chip platforms remain technically demanding, costly, and not yet widely standardized. However, they represent a powerful complement to traditional in vitro and in vivo models, bridging the gap between reductionist systems and clinical reality [34]. Their ability to incorporate patient-derived material further suggests strong potential for personalized medicine and therapeutic discovery in prostate cancer. The first notable use of microfluidics in prostate cancer research was in 2014 with the “Prostate Cancer-on-Chip” model, which simulated the tumor microenvironment by co-culturing prostate cancer cells and stromal fibroblasts in microfluidic channels [63]. This setup allowed continuous signaling between tumor and stroma and showed that conversion of normal fibroblasts into cancer-associated fibroblasts depended on local concentrations of signaling molecules.

### 2.2. Rat in the Development of Drugs for Prostate Cancer Treatment

There are multiple therapeutic approaches for PCa treatment, including surgery, radiotherapy, hormone therapy, immunotherapy, and chemotherapy [13,14,15,16]. The process of developing cancer drugs is lengthy and costly, involving stages such as discovery, preclinical testing, clinical trials across phases one to three, regulatory approval, and post-marketing surveillance. Despite this extensive process, most anti-cancer drug developments face inefficiency, largely due to a lack of efficacy in human patients. Notably, most preclinical studies concentrate predominantly on evaluating drug response, often neglecting essential factors such as pharmacokinetics, pharmacodynamics, toxicity profiles, and drug delivery mechanisms [64,65,66].

**Figure 2:**
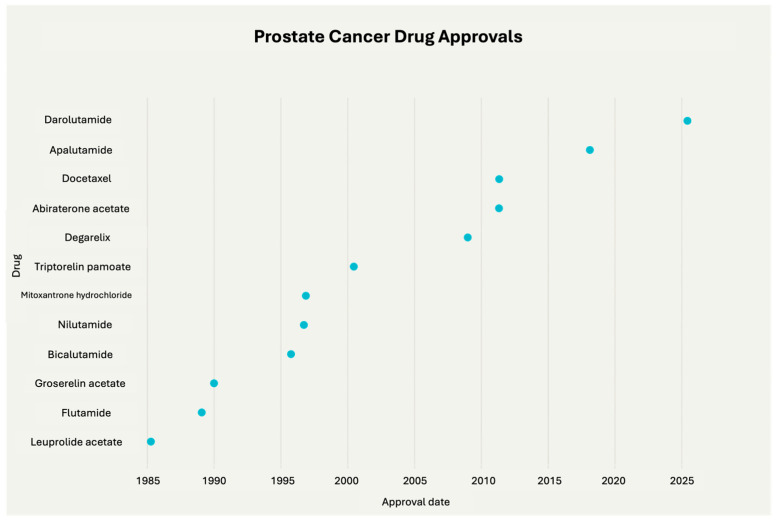
Timeline highlighting the FDA approvals of drugs for prostate cancer treatment over the years. Chart made with Perplexity.

According to the U.S. FDA, there are currently 23 approved drugs available for PCa treatment. The first of these, leuprolide, was approved in 1985, and the most recent, darolutamide, received approval in June 2025, supported by data demonstrating improved outcomes in metastatic castration-sensitive PCa (Figure 2). It is worth noting that the development of these drugs has been greatly supported by studies utilizing various rat strains to investigate drug pharmacokinetics and effects, playing a vital role in preclinical evaluation and translational research [67].

**Figure 3:**
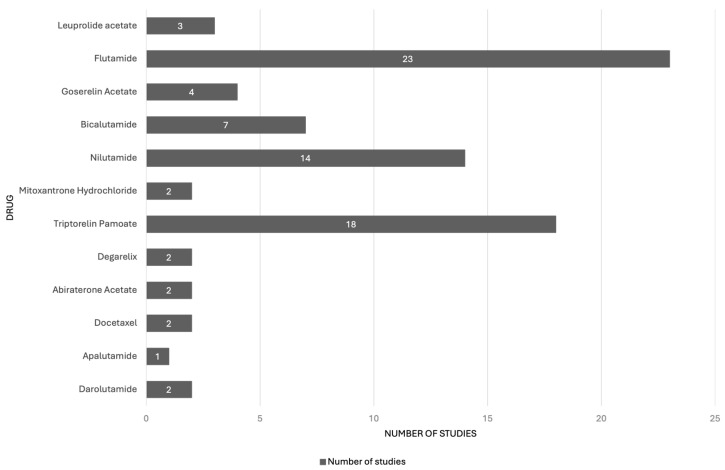
Number of preclinical studies conducted in rats contributing to the development and approval of drugs for prostate cancer treatment.

**Figure 4:**
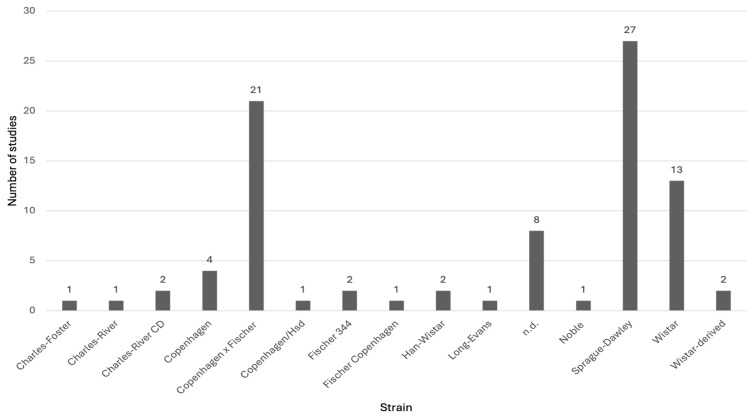
Number of preclinical studies using different rat strains that contributed to the development and approval of drugs for prostate cancer treatment.

A PubMed search was conducted on the 1 October 2025 using the term “rat” in combination with the names of FDA-approved drugs for PCa treatment. A total of 80 studies in rats, conducted before the FDA approval of each drug currently available for prostate cancer treatment, were identified. Most of these studies involved the drugs flutamide (n = 23), triptorelin pamoate (n = 18), and nilutamide (n = 14) (Figure 3). Multiple rat strains were used across these studies, including Charles-Foster, Charles-River, Charles-River CD, Copenhagen, Copenhagen × Fischer, Copenhagen/Hsd, Fischer 344, Fischer Copenhagen, Han-Wistar, Long-Evans, Noble, Sprague-Dawley, Wistar, and Wistar-derived rats. Among these, Sprague-Dawley rats (27 studies) and Copenhagen × Fischer rats (21 studies) were the most employed (Figure 4).

The studies conducted prior to the approval of antiandrogenic and non-antiandrogenic drugs now available for PCa treatment are summarized in Table 1 and Table 2, respectively. Several studies have investigated the effects of drugs in rats without induced PCa. Additionally, some studies used transplanted models of PCa, created by subcutaneous implantation of various PCa cell lines including R3327, R3327-AT-1, R3327-G, R3327-H, and 11095. The animals used varied in age (ranging from 90 days to 15 months) and weight (from 50 g to 400 g). Different administration routes of the drugs were employed, such as subcutaneous, intraperitoneal, oral, and intravenous. Drug dosing frequency also varied, typically administered once or daily over periods ranging from a few days (3 days) to several months (up to 12 months). Only a limited number of studies assessed drug bioavailability, with darolutamide demonstrating good bioavailability. Most studies focused on examining drug impacts on the gonads and accessory glands, as well as serum luteinizing hormone (LH) and testosterone levels. The results consistently showed antiandrogenic effects, characterized by reductions in the weight and volume of the prostate and seminal vesicles, alongside decreased serum LH and testosterone levels, suggesting the beneficial use of these drugs for PCa treatment.

### 2.3. Zebrafish in Prostate Cancer Modelling: Current Status and Future Directions

The zebrafish (*Danio rerio*) has been established as a valuable vertebrate model organism in biomedical research due to its combination of genetic, physiological, and experimental advantages. Its genome shares approximately 71–82% of human disease-related genes, including those regulating cell cycle, tumor suppression, and oncogenic signaling [143]. External fertilization, rapid embryonic development, and the optical transparency of early life stages allow the direct observation of developmental and pathological processes in vivo [144,145]. In addition, the high fecundity of zebrafish and their low maintenance costs, when compared with mammalian species, enable large-scale experimentation that are also aligned with the principles of the 3Rs (Replacement, Reduction, Refinement) [146]. These characteristics, together with the availability of pigment-deficient strains such as Casper, which retain transparency into adulthood [147], have made zebrafish a compelling model that will greatly complement the bridging between cell-based assays and more complex mammalian studies.

In oncology, zebrafish models have been obtained through genetic modification or transplantation, with only rare cases of sporadic tumor formation reported in aged wild-type fish [148,149]. Transgenic and mutagenesis-based strategies enable de novo tumor induction through oncogene overexpression or tumor suppressor gene inactivation [150,151,152], using chemical mutagens (e.g., dibenzo(a,l)pyrene, 7,2-dimethylbenz(a)anthracene, *N*-ethyl-*N*-nitrosourea, *N*-methyl-*N*′-nitro-*N*-nitrosoguanidine), insertional mutagenesis (e.g., transposons, viral vectors), or genome engineering techniques such as CRISPR/Cas9 [153]. These techniques allow the development of tumors that recapitulate the histopathological and molecular features of their human counterparts [154]. Transplantation models, including both allografts and xenografts, are widely used because they enable rapid studies of localized growth, angiogenesis, metastasis, and single-cell tumor–host interactions in real time [155]. Generally, fluorescently labeled tumor cells are microinjected into the yolk sac, perivitelline space, duct of Cuvier, or circulation of embryos at 2–3 d post-fertilization, when adaptive immunity is not yet functional [156]. Because zebrafish lack organs such as lungs, mammary glands, and the prostate [157], xenograft transplantation using commercial or patient-derived cancer cells into embryos or adults is the only viable approach for modelling these tumor types [158,159,160]. The larval stage allows engraftment without immunosuppression, whereas adult models require immune suppression (e.g., gamma radiation, glucocorticoids) or the use of immunodeficient lines (e.g., the prkdc^−/−^, il2rga^−/−^ Casper strain) [161,162,163]. These zebrafish cancer models have been instrumental in evaluating effects on tumor growth and oncogenic pathways. For example, a genetic screen revealed dihydrolipoamide S-succinyltransferase as a target for MYC-driven tumors, leading to clinical testing of its inhibitor devimistat in multiple cancers [164]. Similarly, RNAi-based knockdown experiments in pancreatic cancer xenograft model identified LIMK1/2 as angiogenesis regulators [155].

PCa research has benefited from the use of zebrafish models by adapting xenograft models using both androgen-dependent and androgen-independent human cell lines [165]. LNCaP cells, which require androgen signaling, and lines such as PC-3, DU145, and C4-2, which are androgen-independent, have been used to explore hormone-driven growth, metastatic behavior, and therapeutic responses. In one study, supplementation with testosterone in LNCaP xenografts significantly increased tumor proliferation, an effect reversed by the androgen receptor antagonist enzalutamide, while androgen-independent lines showed no such hormonal modulation [165]. Another study integrated the co-injection of PCa cells with cancer-associated fibroblasts to investigate tumor–stroma interactions, revealing that stromal components can enhance proliferation and micrometastasis, effects that can be mitigated by targeted inhibition of the TGF-β pathway [166]. The zebrafish microenvironment has also been shown to influence epithelial–mesenchymal transition and cancer stem cell phenotypes, underlining its utility for dissecting mechanisms of metastatic initiation [167].

Beyond modelling tumor growth and dissemination, zebrafish also serve as an effective platform for preclinical drug discovery and development (Table 3). Both target-based and phenotype-based screening approaches are feasible [168], facilitated by the small size, permeability of the larvae to waterborne compounds, and compatibility with high-throughput formats [169]. In PCa, zebrafish xenograft models have been used to evaluate several bioactive compounds with distinct mechanisms of action, although the number of studies remains limited compared with other tumor types. For example, pyripyropene O, which targets the YY1–DR5 axis and promotes apoptosis and therefore inhibits proliferation, was shown to inhibit the growth of PC-3 cells in vivo with a zebrafish xenograft model confirming a dose-dependent reduction in tumor burden over 72 h without significant toxicity [170]. Similarly, ilicicolin C, a fungal metabolite that inhibits the PI3K/AKT/mTOR pathway, significantly decreased tumor area in PC-3 cell zebrafish xenografts, indicating suppression of proliferation [171]. Dankasterone A, previously validated in vitro for oxidative stress–mediated cytotoxicity, likewise reduced tumor cell area in vivo, supporting its antiproliferative effects [172]. Kaempferol, a flavonoid with reported activity against both androgen-dependent and -independent PCa cells, inhibited tumor expansion and dissemination of 22Rv1 xenografts over a 6 d exposure period [173]. At a more mechanistic level, the ETS-domain inhibitor VPC-18005 reduced dissemination of ERG-expressing PCa cells in zebrafish compared with controls, while also demonstrating a more favorable toxicity profile than another previously published inhibitor (YK-4-279) [174]. Nevertheless, the number of compounds evaluated in zebrafish PCa models remains limited when compared with other zebrafish models of melanoma, leukemia, or glioblastoma, where compound libraries and mechanistic endpoints have been explored in greater depth [175,176,177,178]. Moreover, in most PCa zebrafish studies to date, mechanistic validation has relied on in vitro data, with in vivo assays limited to measuring tumor size or distribution by fluorescence imaging. This approach underutilizes this model’s capacity to assess additional endpoints such as angiogenesis, invasion dynamics, metastatic spread, or pharmacokinetic–pharmacodynamic relationships [179].

The development of patient-derived xenograft models in zebrafish (zPDXs) represents an even more important evolution of zebrafish use in cancer research. In these models, small quantities of fresh patient tumor tissue are implanted into zebrafish larvae or immunodeficient adults, allowing important aspects such as tumor heterogeneity and microenvironmental interactions to be maintained [180,181]. The main body of zPDX research to date has been in cancers such as lung, breast, colorectal cancer, and leukemia, where strong correlations have been observed between zPDX drug response and clinical outcomes [180,181,182]. PCa applications remain comparatively scarce, limiting the depth of evidence for predictive accuracy in this setting. Nevertheless, given the promising performance of zPDXs in other cancer types, their extension to PCa research is a logical next step although their predictive value in this type of cancer remains largely unvalidated. The capacity of zPDXs to generate therapeutic efficacy data within 4–7 d [183], while requiring minimal tumor material, aligns well with the time-sensitive demands of precision oncology, especially with highly aggressive cancers with limited patient survival. Accordingly, zebrafish should be regarded as early-stage, proof-of-concept models within multi-model preclinical pipelines, while their predictive relevance in prostate cancer remains to be rigorously validated in mammalian models and clinical settings.

## 3. From Bench to Bedside: Challenges in Translating Preclinical Model to Humans

Despite their long-standing value in scientific research, rat models present distinct opportunities and limitations when used as translational systems for prostate cancer studies [184]. Animal models do not replicate the full complexity of human diseases but instead capture specific research relevant aspects and, when properly designed and conducted, provide essential insights into disease biology and therapeutic development [185]. Their scientific relevance depends on rigorous experimental design and on their capacity to meaningfully inform disease mechanisms [186]. Consequently, high quality translational research requires a clearly defined research question, and the selection of a model demonstrably fits its intended purpose [185]. The translation of findings from rat models to humans in PCa research faces substantial challenges, many of which stem from the specific rat strains employed and from interspecies anatomical, physiological, and molecular differences [27,30]. In humans, the prostate is organized as a single gland, whereas in rats it is divided into multiple lobes, a structural divergence that influences disease progression and complicates direct comparisons between species [187]. Nevertheless, rat and human prostate carcinogenesis share several critical molecular pathways, reinforcing the value of rat models for elucidating disease mechanisms and for advancing preventive and therapeutic interventions [33,187]. A further challenge inherent to rat PCa models is their typically long latency period, low spontaneous tumor incidence, and limited metastatic capacity [184,188]. To overcome these constraints, researchers often adopt strains or protocols specifically designed to enhance tumor yield and experimental consistency. Chemically induced models, such as MNU combined with testosterone in Lobund-Wistar or Fischer F344 rats, can achieve high tumor incidence [189,190]. Similarly, transgenic lines carrying prostate-specific oncogenic drivers, such as SV40 T antigen, reliably develop prostate adenocarcinoma with almost complete penetrance by roughly 25 weeks of age [191]. These refined systems help compensate for the inherently slow and infrequent tumorigenesis of standard rat models and thereby expand their relevance for investigations. Selecting a rigorously validated and predictive animal model is fundamental to ensuring that the experimental system is fit-for-purpose and capable of addressing the defined clinical or biological question [184,185,186]. When the model is appropriately matched to the research objective, it substantially enhances the translational strength of the findings and increases their relevance for human prostate cancer [184,185].

In addition to the previously discussed limitations inherent to mammalian models, zebrafish models also face several obstacles that limit their effective contribution to translational drug-development efforts directed toward human clinical use, particularly in the PCa research. Most importantly, zebrafish lack a prostate and associated reproductive structures, restricting disease modelling to xenograft-based approaches and preventing the study of spontaneous or hormonally driven carcinogenesis [157]. Furthermore, zebrafish must be maintained at temperatures (typically 26–28 °C in standard aquatic systems) considerably lower than human physiological conditions, and this difference can alter human cancer cell metabolism, proliferation rates, gene expression profiles, and drug responses [183]. Efforts to mitigate these issues have led to the development of immunodeficient strains capable of tolerating temperatures up to 37 °C, such as the *prkdc*^−/−^, *il2rga*^−/−^ Casper line, which tolerates 37 °C and allows more physiologically relevant engraftment [163]. Even so, these fish must be maintained under demanding husbandry conditions, including continuous antibiotic exposure, sterilized water with frequent replacement, and strict, gradual thermal acclimation, and still display alterations in tumour growth dynamics, histological features, stress physiology, and long-term viability, with overall survival remaining reduced under these conditions [163]. Further limitations come from the methodological setup of zebrafish assays, many of which are short term and use surrogate endpoints like fluorescent tumour area or relative dissemination. While such results may be valuable for rapid screening, they provide limited insights into systemic toxicity, long-term remission, pharmacodynamics, tumour evolution and/or mechanisms of resistance; such outcomes are more readily evaluated in mice and rat through histopathology, gene expression analyses, multi-organ assessment, and longitudinal follow-up. Drug administration by immersion presents an additional challenge, as it introduces uncertainties in dose delivery, absorption, and pharmacokinetics, thereby limiting the extrapolation to clinically relevant oral or intravenous routes in humans [192]. Beyond these factors, substantial heterogeneity in experimental design, including variation in cell lines, injection sites, imaging modalities, quantification strategies, and endpoints, reduces reproducibility and hinders cross-study comparisons. In PCa specifically, the predictive value of zebrafish models remains to be fully validated. Although several proof-of-concept studies have demonstrated used PCa zebrafish xenografts [165,174], the scarcity of large datasets correlating zebrafish responses with PCa patient outcomes will continue to limit regulatory implementation. Standardization of xenograft and zPDX methodologies, combined with systematic benchmarking against mammalian and clinical data, is therefore essential for strengthening translational reliability. Nonetheless, zebrafish are expected to play an increasingly important role in oncology and prostate cancer research, particularly as xenograft and zPDX methodologies become more refined and standardized. Ultimately, the integration of zebrafish within coordinated multi-model pipelines, rather than their use in isolation, represents the most realistic path forward for overcoming these obstacles and improving the success rate of cancer drug development, as has been increasingly advocated across multiple disease areas [193,194,195]. Advances in imaging technologies, genetic engineering, and the development of immunodeficient strains have strengthened their potential as efficient intermediate models between in vitro systems and mammalian preclinical trials [169]. Yet, the true translational impact of zebrafish still depends on how effectively they can complement and inform preclinical studies clinical studies, especially in areas such as pharmacokinetics, toxicity, and therapeutic resistance. Thus, zebrafish models should be regarded as complementary intermediate platforms within a tiered preclinical strategy, rather than as standalone predictors of clinical success in prostate cancer.

## 4. Conclusions

PCa remains a major global health challenge. Notwithstanding the advances achieved in the domains of molecular and imaging science, the clinical translation of research findings continues to be constrained by the limitations of current preclinical models. PCa modelling has evolved significantly over the last century, progressing from simple cell-based models to more complex systems. In vitro cancer cell lines have been critical in elucidating pathways and conducting drug screens but lack a full tumor microenvironment context. In vivo models, namely rodents, are essential for studying the physiological relevance of PCa carcinogenesis and therapy response. The employment of complementary models, such as CAM and zebrafish embryos, expands experimental options.

Rats have played a foundational role in the development of drugs for prostate cancer, providing essential data on pharmacokinetics and drug safety that supported the approval of currently available therapies. Their physiological and genetic similarities to humans, and well-established induced and transgenic models, make them indispensable for preclinical investigation. Zebrafish has emerged as a powerful complementary system, offering rapid, high-throughput platforms for studying tumor behavior and early-stage drug responses. Despite these advantages, zebrafish PCa models—restricted to xenografts—are constrained by anatomical differences, thermal incompatibility with human cells, short-term assay design, and limited validation against clinical outcomes.

The integration of traditional mammalian models with emerging systems, such as zebrafish, offers a more comprehensive strategy for research into PCa. By leveraging the strengths of each model, researchers can achieve a more comprehensive understanding of the disease mechanisms, enhance the predictive accuracy of candidate therapeutics, and ultimately improve the efficiency of translating preclinical findings into effective patient-centered treatments.

This review provides an overview of the evolution and variety of PCa models, emphasizing their complementary roles in translational research. It highlights the significant contributions of rat models to drug development and FDA approval, while also exploring the growing potential of zebrafish models in advancing precision oncology, all while addressing their current biological and methodological limitations.

## Figures and Tables

**Table 1:** Studies performed in rats previously to the FDA approval of antiandrogenic drugs currently used for prostate cancer treatment.

Drug/Approval Date	Year	Strain	Model	Age/Weight	Administration Route	Dose	Frequency	Duration	Effects	Reference
Leuprolide acetate 9 April 1985	1983	Noble	Pr tumor implant (s.c.)	290–319 g	s.c.	20, 50 μm	2×/d	12 wks	↓ Pr and SV weight, and LH and testosterone	[68]
	1984	Copenhagen × Fischer	R3327-G Pr tumor (s.c.)	200–250 g	s.c.	1, 50, 100 μm/kg	5, 7×/wk	2 wks	↓ testis and tumor weight; ↓ testosterone	[69]
	1984	Sprague-Dawley		250 g	s.c.	200 ng	1×/d	14 d	↓ Pr and SV weight	[70]
Flutamide 27 January 1989	1974	Charles-River CD		250–300 g	p.o./i.p.	15 mg/kg	1×/d	3, 7 d	↓ Pr and SV weight	[71]
	1974	Sprague-Dawley		350–400 g	i.p.	1, 10 μm	Once	once	Anti-androgenic effect	[72]
	1976	n.d.		150–200 g	p.o./i.p.	25 mg/kg	1×/d	30 d	↓ Pr and SV weight	[73]
	1977	Wistar		300–400 g	i.p.				↓ androgens binding	[74]
	1979	Charles-River CD		50–65 g	p.o.		Once		Anti-androgenic effect	[75]
	1980	Long-Evans		70 d	s.c.	5 mg	1×/d	5 d	↓ Pr and SV weight	[76]
	1981	Charles-River		200 g	s.c.	33, 100, 300 μm	once		↓ Pr and SV weight	[77]
	1982	Charles-Foster		220 g	i.p.	0.25, 2.5, 5 mg/100 g b.w.	1×/d	7 d	↓ Pr and SV weight	[78]
	1983	Wistar		120 d	s.c.	20 mg/kg	1×/d	14 d	Anti-androgenic affect	[79]
	1984	Sprague-Dawley		275–310 g	s.c.	25 mg/kg	1×/d	7 d	↓ Pr weight	[80]
	1984	Sprague-Dawley		250 g	s.c.	15 mg/kg	1×/d	7 d	↓ Pr and SV weight, and LH	[81]
	1984	Sprague-Dawley		250–275 g		15 mg/d	1×/d	20 d	Anti-androgenic effect	[82]
	1985	Copenhagen × Fischer	R3327-H Pr tumor (s.c.)		s.c.	25 mg/kg	1×/d	60 d	↓ tumor volume; Pr and testes weights	[83]
	1986	Wistar		300 g	s.c.	5, 25 mg/kg	1×/d	14 d	↓ Pr and SV weight	[84]
	1986	Sprague-Dawley		275–300 g	s.c.	10 mg/kg	1×/d	14, 28 d	↓ Pr and SV weight	[85]
	1987	Han-Wistar		250 g	s.c.	10 mg/kg	1×/d	1–12 wks	↓ Pr and SV weight	[86]
		Han-Wistar		350 g	s.c.	100 mg/d	1×/d	2 wks	Study of drug pharmacokinetics	[86]
	1987	Wistar-derived				1, 5, 25 mg/kg	1×/d	14 d	↓ growth of the SV and ventral Pr; did not influence hormones	[87]
	1987	Wistar		60–85 d	s.c.	25 μm/kg	1×/d	10 d	↓ Pr and SV weight	[88]
	1988	Sprague-Dawley		250–275 d		15 mg/d	1×/d	8 d	↓ Pr and SV weight	[89]
	1988	Copenhagen × Fischer	R3327 Pr tumor (s.c.)		p.o.	15 mg/kg	1×/d	4 wks	↓ tumor size	[90]
	1988	Wistar		250–350 g	s.c.	10 mg	3×	4 d	Suppression of androgenic mechanisms	[91]
	1988	Sprague-Dawley		225–250 g		5 mg	2×/d	7 d	↓ Pr weight	[92]
	1988	n.d.		170–190 g	p.o.	5 mg/kg	1×/d	4 d	Inhibited growth of the SV and ventral Pr gland; increase testosterone and LH	[93]
Bicalutamide 4 October 1995	1987	Wistar-derived			s.c.	1, 5, 25 mg/kg	1×/d	14 d	Inhibited growth of the SV and ventral Pr gland; did not influence hormone	[87]
	1988	n.d.		170–190 g		25 mg/kg	1×/d	7 d	Anti-androgenic activity (selective anti-androgen)—reduced testosterone and LH	[93]
	1989	Wistar		200–300 g	i.v.	25 mg/kg	1×/d	28 d	Anti-androgenic activity (selective anti-androgen)—reduced testosterone and LH	[94]
	1989	Sprague-Dawley		200 g	p.o.	50 mg/kg	1×/d	14 d	Inhibited ventral Pr and SV weight gain; minimal effects on testosterone	[95]
	1993	Copenhagen × Fischer	R3327-G Pr tumor (s.c.)	8 wks	p.o.	10 mg/kg	1×/d	15 d	↓ Pr and SV weights	[96]
		Copenhagen × Fischer	R3327-G Pr tumor (s.c.)	8 wks	s.c.	20 mg/kg	3×/wk	4 wks	↓ tumor growth	[96]
	1994	Wistar		200 g	s.c.	1, 3, 10 mg/d	1×/d	14 d	↓ Pr weight	[97]
	1995	Fischer 344	DMBA (50 mg/kg, s.c., 10 wks) + testosterone tube (20 mg; 60 wks)	6 wks	p.o.	30 mg/kg	3×/wk	20, 39 wks	Inhibited the development of PCa	[98]
Nilutamide 19 September 1996	1981	Sprague-Dawley		225–250	s.c.	5 mg/d	1×/d	2 wks	↓ Pr and SV weight	[99]
	1984	Sprague-Dawley		200 g	s.c.	0.2, 1, 5, 10 mg/d		14 d	↓ ventral Pr and SV weight	[100]
	1986	n.d.		Adult	p.o.	10 mg/kg	1×/d	8 d	↓ Pr weight	[101]
	1986	Sprague-Dawley		275–300 g	s.c.	10, 20 mg/kg	1×/d	14, 28 d	↓ Pr and SV volume	[85]
	1987	n.d.		Adult	p.o.	20 mg/kg	1×/d	5, 10, 15 d	↓ Pr weight	[102]
	1988	Sprague-Dawley		220–250 g	p.o.	20 mg/kg	1×/d	15 d	↓ Pr and testis weight	[103]
	1988	Copenhagen			s.c.	8 μm	Once	6 wks	↓ Pr, SV and testis weight	[104]
	1988	Wistar		250–350 g	s.c.	3 mg/kg	3 times	4 d	Suppression of androgenic mechanisms	[91]
	1988	Copenhagen			s.c. implant	8 μm	Once	3 d	↓ testosterone	[105]
	1990	Sprague-Dawley		61 d	p.o.	0.5, 5 mg	1×/d	30 d	Inhibited the growth of accessory glands	[106]
	1990	Sprague-Dawley		170–200 g	p.o.	5, 10 mg/kg	1×/d	15, 30 d	↓ testis and accessory glands weight; increased testosterone	[107]
	1991	n.d.			i.v./p.o.	10 mg/kg	Once	24 h	Plasma decay of nilutamide is very slow	[108]
	1991	n.d.			p.o.	0.4, 2, 10 mg/kg	1×/d	15 d	↓ Pr weight	[109]
	1991	n.d.		120 d	i.p.	5 mg/100 g b.w.			Increased nuclear androgen receptors	[110]
Triptorelin pamoate 15 June 2000	1981	Sprague-Dawley			s.c.	10 μm/d	1 ×/d	5 d	↓ Pr and Sv weights	[111]
	1981	Fischer 344	11095 Pr tumor (s.c.)	100–120 g	s.c.	25 μm	1×/d	14–21 d	↓ tumor growth, and testosterone	[112]
		Copenhagen × Fischer	R3327 Pr tumor (s.c.)		s.c.	25 μm	1×/d	42 d	↓ tumor growth, and testosterone	[112]
	1983	Copenhagen × Fischer	R3327 Pr tumor (s.c.)		s.c.	25 μm	2×/d	21 d	↓ tumor growth, and testosterone	[113]
	1984	Wistar	R3327 Pr tumor (s.c.)	180–200		25 μm	2×/d	21 d	↓ tumor weight	[114]
	1984	Copenhagen × Fischer	R3327 Pr tumor (s.c.)		s.c.	25 μm	2×/d	28 d	↓ tumor weight	[115]
		Copenhagen × Fischer	11095 Pr tumor (s.c.)						↓ tumor weight	[115]
	1984	Copenhagen × Fischer	R3327-H Pr tumor (s.c.)		s.c.	12.5, 25 μm	1×/d	30 d	↓ tumor weight and testosterone	[116]
	1985	Copenhagen × Fischer	R3327-H Pr tumor (s.c.)			25 μm/d		14 d	↓ activity of plasminogen activators (inhibition of fibrinolytic process)	[117]
	1985	Copenhagen × Fischer	R3327-H Pr tumor (s.c.)		i.m.	25 μm/d		30, 60 d	↓ tumor and testis weight and volume	[83]
	1985	Copenhagen × Fischer	R3327-H Pr tumor (s.c.)		i.m.	25 μm/d		60–100 d	↓ tumor and testis weight and volume, and testosterone	[118]
	1986	Sprague-Dawley		300 g		Microcapsules			↓ testosterone	[119]
	1986	Copenhagen × Fischer	R3327-H Pr tumor (s.c.)		i.m.	25 μm/d		60, 70, 105 d	↓ Pr weight; and testosterone and LH	[120]
	1987	Copenhagen × Fischer	R3327-H Pr tumor (s.c.)		i.m.	25 μm/d		45, 70, 135 d	↓ tumor weight and volume	[121]
	1987	Copenhagen × Fischer	R3327-H Pr tumor (s.c.)		s.c.	25 μm	2×/d	70, 83 d	↓ tumor weight	[122]
	1988	Copenhagen × Fischer	R3327-H Pr tumor (s.c.)	351 g	s.c.	25 μm	2×/d	30 d	↓ tumor weight and volume; and tumor proliferation	[123]
	1988	Copenhagen × Fischer	R3327-H Pr tumor (s.c.)		i.m.	25 μm/d		83 d	↓ Pr tumor volume	[124]
	1988	Sprague-Dawley		325–350 g		1 μm/d		10 d	↓ ventral Pr and SV weights; and testosterone and LH	[125]
	1988	Sprague-Dawley		225–250 g	s.c.	5 μm	1×/d	14 d	↓ Pr weight	[126]
	1991	Copenhagen × Fischer	R3327-H Pr tumor (s.c.)		s.c.	25 μm/d		8 wks	↓ number of Ag-NORs in the tumors	[127]
	1992	Sprague-Dawley			s.c.	50 μm	Once		Increased LH	[128]
	1994	Copenhagen	R-3327-AT-1 Pr tumor (s.c.)	300–320 g		100 μm/d		2 wks	Inhibition of tumor growth	[129]
Degarelix 24 December 2008	2004	Sprague-Dawley			s.c.	0.4, 1.0, 1.5 mg/kg	Once	Once	↓ LH	[130]
	2007	Copenhagen/Hsd	R3327-H Pr tumor (s.c.)	7–8 wks	s.c.	1 mg/kg	1×/mo	2, 12 mo	Inhibited tumor growth	[131]
Abiraterone acetate 28 April 2011	2003	Wistar		220–240 g	p.o.	50 mg/kg	1×/d	3 d	↓ Pr and SV weight	[132]
	2003	Sprague-Dawley		2–3 mo	i.p.	5 mL/kg	1×/d	14 d	↓ Pr and SV weights, and testosterone	[133]
Apalutamide 14 February 2018	2017	Sprague-Dawley			p.o.	10 mg/kg	Once	Once	Study of drug pharmacokinetics	[134]
Darolutamide 3 June 2025	2020	Sprague-Dawley		215 g	p.o.	10 mg/kg			Good bioavailability	[135]
		Sprague-Dawley			i.v.	1.0 mg/kg			Good bioavailability	[135]
	2025	Sprague-Dawley			p.o./i.v.	0.5, 1.0 mg/kg	Once	Once	Good bioavailability	[136]

b.w.: body weight; d: day(s); i.m.: intramuscular; i.p.: intraperitoneal; i.v.: intravenous; LH: luteinizing hormone; mo: month(s); n.d.: not determined; p.o.: per os; PCa: prostate cancer; Pr: prostate; s.c.: subcutaneous; SV: seminal vesicle; wks: weeks; ↓: decreased.

**Table 2:** Studies performed in rats previously to the FDA approval of non-antiandrogenic drugs currently used for prostate cancer treatment.

Drug/Approval Date	Year	Strain	Model	Age/Weight	Administration Route	Dose	Frequency	Duration	Effects	Reference
Goserelin acetate 29 December 1989	1987	Copenhagen	R3327-H Pr tumor (s.c.)	>90 d	s.c. implant			10 wks	↓ Pr and SV weight, and testosterone, restoration of normal elements in neoplastic cells	[137]
	1987	Wistar		12–15 mo	s.c. implant	1 μm/h		28 d	↓ Pr and SV weight; testosterone not changed	[138]
	1988	Wistar		200–220 g	s.c.	0.9 mg	1×/d	4, 10, 17 d	↓ Pr and testes weight; LH and testosterone	[139]
	1989	Wistar		300–500 g	s.c.	3.6 mg/depot	1×/mo	2–24 wks	Testis suppression	[140]
Mitoxantrone hydrochloride 13 November 1996	1986	Copenhagen × Fischer	R3327-H Pr tumor (s.c.)		i.v.	0.25 mg/kg	1×/wk	3 wks	↓ Pr, SV and tumor volume	[120]
	1987	Copenhagen × Fischer	R3327-H Pr tumor (s.c.)		i.v.	0.25 mg/kg	Every 3 wks	10, 15 wks	↓ tumor volume and weight	[121]
Docetaxel 3 May 2011	2008	Fischer Copenhagen	R3327 Pr tumor (s.c.)	12 wks	i.p.	10 mg/kg	Once		↓ tumor growth	[141]
	2010	Sprague-Dawley			i.v.	10 mg/kg	Once		Study of drug pharmacokinetics	[142]

d: day(s); i.p.: intraperitoneal; i.v.: intravenous; LH: luteinizing hormone; mo: month(s); Pr: prostate; s.c.: subcutaneous; SV: seminal vesicle; wk: week; wks: weeks; ↓: decreased.

**Table 3:** Studies in the literature using zebrafish PCa xenograft models in drug discovery research.

Strain	Cell Line(s)	Drug	Dose	Duration	Effect	Reference
Wild-type	VCaP, PNT1B-ERG	VPC-18005	1–10 μM	5 d post-injection	↓ dissemination (20–30%)	[174]
Wild-type	VCaP, PNT1B-ERG	YK-4-279	1–10 μM	5 d post-injection	↓ dissemination (40–60%)	[174]
Wild-type	PC-3	Dankasterone A	0.3125–2.5 μM	72 h post-injection	⛔ xenograft growth	[172]
Wild-type	PC-3	Ilicicolin C	10^−8^–10^−7^ M	72 h post-injection	⛔ xenograft growth	[171]
Wild-type	PC-3	Pyripyropene O	2.5–10 μM	72 h post-injection	⛔ xenograft growth	[170]
Transgenic (s843Tg/+)	22Rv1	Kaempferol	10–40 μM	6 d post-injection	⛔ xenograft growth	[173]

↓: decreased; ⛔: inhibition; PCa: prostate cancer.

## Data Availability

No new data were created or analyzed in this study. Data sharing is not applicable to this article.

## Abbreviations

The following abbreviations are used in this manuscript:

CAM	Chorioallantoic membrane
CDX	Cell-derived xenograft
PCa	Prostate cancer
PDX	Patient-derived xenograft
zPDX	Zebrafish patient-derived xenograft
